# Research Stakeholders’ Views on Benefits and Challenges for Public Health Research Data Sharing in Kenya: The Importance of Trust and Social Relations

**DOI:** 10.1371/journal.pone.0135545

**Published:** 2015-09-02

**Authors:** Irene Jao, Francis Kombe, Salim Mwalukore, Susan Bull, Michael Parker, Dorcas Kamuya, Sassy Molyneux, Vicki Marsh

**Affiliations:** 1 Health Systems and Research Ethics Department, Kenya Medical Research Institute Wellcome Trust Research Programme, Kilifi, Kenya; 2 Ethox Centre, Nuffield Department of Population Health, Oxford University, Oxford, United Kingdom; 3 Centre for Tropical Medicine and Global Health, Nuffield Department of Medicine, Oxford University, Oxford, United Kingdom; London School of Hygiene and Tropical Medicine, UNITED KINGDOM

## Abstract

**Background:**

There is increasing recognition of the importance of sharing research data within the international scientific community, but also of the ethical and social challenges this presents, particularly in the context of structural inequities and varied capacity in international research. Public involvement is essential to building locally responsive research policies, including on data sharing, but little research has involved stakeholders from low-to-middle income countries.

**Methods:**

Between January and June 2014, a qualitative study was conducted in Kenya involving sixty stakeholders with varying experiences of research in a deliberative process to explore views on benefits and challenges in research data sharing. In-depth interviews and extended small group discussions based on information sharing and facilitated debate were used to collect data. Data were analysed using Framework Analysis, and charting flow and dynamics in debates.

**Findings:**

The findings highlight both the opportunities and challenges of communicating about this complex and relatively novel topic for many stakeholders. For more and less research-experienced stakeholders, ethical research data sharing is likely to rest on the development and implementation of appropriate trust-building processes, linked to local perceptions of benefits and challenges. The central nature of trust is underpinned by uncertainties around who might request what data, for what purpose and when. Key benefits perceived in this consultation were concerned with the promotion of public health through science, with legitimate beneficiaries defined differently by different groups. Important challenges were risks to the interests of study participants, communities and originating researchers through stigmatisation, loss of privacy, impacting autonomy and unfair competition, including through forms of intentional and unintentional 'misuse' of data. Risks were also seen for science.

**Discussion:**

Given background structural inequities in much international research, building trust in this low-to-middle income setting includes ensuring that the interests of study participants, primary communities and originating researchers will be promoted as far as possible, as well as protected. Important ways of building trust in data sharing include involving the public in policy development and implementation, promoting scientific collaborations around data sharing and building close partnerships between researchers and government health authorities to provide checks and balances on data sharing, and promote near and long-term translational benefits.

## Background

Support for the idea that data from public health research should be made more widely available within the scientific community has recently gathered momentum. Throughout the 2000s, a series of high profile statements supporting increased research data sharing were made by international scientific consortia, funders and standard setters [1]. More recently, requirements for research data sharing have gained a higher profile in science funding [2–5] and publication [6–8]; 88% of the fifty research journals with highest impact factors have statements on the importance of sharing data [9].

The debate around data sharing is complex [10] with wide recognition that the type of data, who has access and how data will be used have the potential to generate context-specific ethical issues [9, 11–13]. Ethical challenges relate to the potential for competing interests to emerge between and amongst different research stakeholders. Alongside scientific utility of a given dataset, key challenges relate to the interests of originating researchers (those who generate data), research participants and study communities. These and other relevant stakeholders—including funders, regulators, publishers of journals, the pharmaceutical industry and wider publics—may have interests that intersect within and across groups [12]. For originating researchers, the main issues include maintaining first rights to publish, risks of intentional or unintentional misrepresentation of data and the resources needed to make data available [1, 9, 11, 13, 14]. For study participants and communities, important challenges include maintaining privacy, ensuring that future use of data is not harmful, and understanding how individual consent should be sought [13, 15–17].

Fairness has emerged as a particular concern for global data sharing when studies are conducted in low-to-middle income countries (LMIC), given structural inequities in access to health services (for primary communities) and scientific resources in these settings (for originating researchers) [1, 11, 13, 18]. Originating researchers in LMICs often invest greater resources and experience more challenges in data collection, analysis and publication than those from better resourced parts of the world. Since high income country researchers are also likely to be able to analyse and publish data more quickly than some in LMICs, data sharing risks undermining career development for LMIC researchers and widening gaps between those in more and less well-resourced settings. As has also been argued for genomics research [19], scientific capacity development in LMICs is key to strengthening the relevance of research in these areas of the world, and countering increasing global health inequities [20]. Responses to the challenge that global data sharing may undermine LMIC researchers have included the now well-established use of embargo periods for data release [1, 16], calls for more collaboration between researchers with differing access to scientific resources, and recognition of the role of scientific capacity building as a means of addressing global inequities [1, 11, 13].

Given the need for public support, trust and well-founded confidence in scientific enterprise, there is increasing recognition that public opinion should inform locally responsive research policies, including on data sharing [12, 21]. Relatively little empirical research has examined these perspectives, particularly from LMICs. This paper draws on findings from a qualitative study in Kenya undertaken as part of a multi-country project set up to address this gap through the Public Health Research Data Forum (PHRDF) [22] and involving Kenya, South Africa, India, Thailand and Vietnam. The current paper describes Kenyan research stakeholders’ perceptions of benefits and challenges in sharing data and the emerging importance of trust at individual and institutional levels. Findings on recommendations for informed consent and governance for data sharing in Kenya are reported separately [23], alongside outputs from other country sites involved in the PHRDF project [10, 24–27].

## Methods

### Study area

This study was conducted at the Kenya Medical Research Institute (KEMRI) Wellcome Trust Research programme (KWTP), an international centre based in Kilifi County hospital on the coast of Kenya. The ‘community’ referred to in this paper is the population of 260,000 residents in the hospital’s main catchment area, and included in a Health and Demographic Surveillance Survey (KHDSS) supporting research and local health service delivery [28]. This mixed community includes some of the most-poor areas of Kenya (primarily rural) [29], county government headquarters and a thriving university. The predominant ethnic group is Mijikenda [30]. Main livelihoods are subsistence farming and fishing, with an emerging urban service industry.

The KWTP was established in 1989 as an active collaboration between researchers in the progamme and government County Health managers in Kilifi. As an active multidisciplinary research programme, planning and implementation of studies are closely tied to County health policy. For example, the research programme strategically supports County hospital managers to ensure that the County Hospital consistently provides good standards of care to all in-patients, through providing resources such as additional staff, equipment, infrastructure and medicines where needed. Many studies within the KWTP involve residents in the broad catchment area of Kilifi County Hospital, including around 260,000 people. The research programme has an active and multi-faceted community engagement programme [31] within which consultation activities draw on a network of approximately 200 KEMRI community representatives (KCRs) across this area. KCRs are ‘typical’ community members selected by and from groups of villages at public meetings to support interactivity for a three year period, and participate in annual workshops on research-related topics. A full description of KWTP, community engagement and the surrounding community are given elsewhere [32].

### Study participants

Sixty participants were purposively selected to reflect varying degrees of research experience. These included early-career to senior scientists (12 individuals); research-based health providers (2); programme front-line staff, including community facilitators (5) and field workers (11); local administrative leaders (6) and community representatives (KCRs) (24). Amongst scientists, 8 were Kenyan nationals and 4 UK nationals. All other study participants were Kenyan nationals. Field workers are group of staff who support research activities, including information-giving about studies, seeking informed consent and collecting data [33]. Field workers and community facilitators routinely participate in training on research methods and ethics, originate from the community and have at least 12 years of formal education. Within groups, participants were selected purposively to reflect diversity in age, gender and—for KCRs—educational status, religion and residency in urban or rural settings. Table 1 summarises participants’ characteristics.

**Table 1:** Characteristics of participants. See also [2 3]

Participants	Total	Age range in years	Men:Women	Education range in years	Religion: Muslim	Religion: Christian	Religion: Other/none
**Research staff**	14	30–59	10:4	5 to 30	3	8	3
**Staff: Fieldworkers**	11	28–45	6:5	12 to 16	0	11	0
**Staff: Community facilitators**	5	30–51	3:2	12 to 18	1	4	0
**Assistant chiefs**	6	33–50	3:3	12 to 18	1	5	0
**KEMRI Community Representatives**	24	26–81	12:12	None (1); informal (1); More than 8 (primary) (10); more than 9 (secondary) (18)	2	22	0

### Data collection

Data were collected between January and June 2014. As a consultation activity, the study employed a deliberative approach, including sharing information to the extent needed to support informed discussion, building on existing knowledge as far as possible, and using probes to explore views on ethical issues. This approach was developed to facilitate debate around unfamiliar complex topics [34, 35], drawing on principles in deliberative forms of ethics [36] and related forms of deliberative consultation [21, 37].

In-depth interviews lasting one to one and a half hours were held with researchers and health providers, drawing on experiences of data sharing. Front-line staff, assistant chiefs and KCRs participated in extended small group discussions, including a case-study and vignette of data sharing from a KHDSS and hospital-based clinical surveillance database, supported by visual aids. The vignette and question guide content are included as a supplementary file (S1 Document). Interviews and discussions were held in Kiswahili, Kigiriama (local language) or English depending on the participants’ choices. Tools used for in-depth interviews (S2 Document) and group discussions (S3 Document) are included as supplementary files.

Eight group discussions were held, each including four to six people and lasting about four hours. In community stakeholder discussions, information-sharing included explaining the potential benefits of sharing research data as an opportunity for more to be learned from data collected during a study through new research, without additional investment of time and other resources. Within a week of each KCR group discussion, 30–45 minute individual structured interviews were held at home with three to four participants to assess the stability of views over time (total 14 participants). Individuals were selected for follow up to reflect differences in attitudes to data sharing, including some who made few contributions to discussions. For group discussions, an experienced note taker kept detailed records, and facilitators held debriefing sessions. Emerging findings were noted and used to inform on-going topic guide development. All interviews and discussions were audio recorded, transcribed and translated into English where needed.

### Data analysis

Data analysis used a Framework Analysis approach [38], using themes from topic guides and emerging from the data. Framework analysis, developed to support social policy research, allows analysis around known and emerging themes, with the maintenance of narrative integrity within the data. Steps include i) familiarisation, ii) thematic analysis to develop a coding structure, iii) indexing or coding of data and iv) charting to support comparisons across the data. Transcripts were read in-depth for familiarisation, discussed between facilitators, and an initial coding framework developed from separate close reading of three transcripts by IJ and VM. The data were then coded using Nvivo 10 software (QSR International). Analysis charts were developed to collate individual and group level views under broad themes relating to perceptions of benefits, challenges and factors influencing acceptability, capturing emerging issues. Separate charts were developed to describe the dynamics of group discussions (for example, changes of opinion over time). Analysis was conducted mainly by IJ and VM, supported by other authors, through an iterative process including cross-checking of coding and analysis charts.

Throughout data collection and analysis, facilitators recognised and aimed to minimise potential biases [38]. This included care in the way information was shared, questions asked, group discussions managed and analysis and interpretation undertaken. For example, a key issue was that alignment of facilitators with the research programme might limit criticism of institutional policies, including for data sharing. Strategies included maintaining a neutral position on the topic under discussion, ensuring positive and negative implications were discussed, and emphasising the role of the consultation in informing future policy. IJ, SMw and FK are experienced facilitators who originate from the community, and were primarily responsible for facilitation of group discussions; VM was primarily responsible for individual interviews. VM, DK and SM have lived in Kilifi for more than 18 years.

### Ethical review

The study was approved by KEMRI Scientific Steering and Ethical Review Committees in Kenya. All participants gave written informed consent for participation.

## Findings

Perceptions of the importance and challenges of sharing research data are described across interviews, group discussions and follow up interviews; attitudes emerged over the course of discussions as a reflection of the way these were perceived and balanced against each other.

There was considerable variation in levels of familiarity with research, research data and data sharing between, and within, more and less research-experienced stakeholder groups. As described in the earlier section, the methods developed for exploring these issues were similarly varied, to take account of these difference. Many community representatives (KCRs) and assistant chiefs were initially unaware that researchers might share data beyond the research team. Although more time was spent in these groups exploring and building understanding of research data and data sharing, some challenges remained in reaching a common understanding of the nature of data, its variability, and its use outside the context in which it was collected. In our analysis, we draw on views expressed and the way these changed over the course of discussions. For example, where concerns raised at the outset of a discussion were largely addressed through more in-depth explanations (for example, around risks of stigmatisation and processes of individual anonymisation), we learned about issues likely to emerge in the absence of much supporting information; and the way these might change with greater understanding. A particular issue, needing repeated exploration, was the distinction between primary and secondary use of research data.

During discussions and interviews, study participants’ views drew on a variety of sources, including real life experiences of data sharing, awareness of the literature and hypothetical situations. Many researchers and some community facilitators had direct experience of sharing data as originators, and some as requestors. For this group, discussions often moved freely between real-life and hypothetical situations, without key differences emerging between these. For less research-experienced participants, their views were based on discussion of hypothetical situations, including the case studies presented.

### Importance of data sharing

Researchers, health providers and community facilitators described concerns and limitations, but expressed universal support for sharing research data, with a majority articulating strongly positive views. The main benefit seen was a potential to support science in fundamental ways, including ensuring the reproducibility, integrity and transparency of science and potentially improving the quality of data. Data sharing was often seen as an essential component of good science, particularly by more experienced researchers. At a minimum, many expressed strong support for journals’ policies on sharing datasets underpinning analyses.

KCRs and field workers were generally more cautious, to some extent reflecting a process of gaining understanding of the benefits and processes involved but also underlining continuing concerns about the need for local benefits of data sharing and to protect the rights and interests of data subjects. Overall, one person (R1FGD8, KCR) remained consistently negative towards data sharing based on concerns about potential for misuse. Across KCRs and field worker groups, many participants supported data sharing on the basis of a potential to maximise health benefits of research. This argument was most strongly supported where translational health benefits of data sharing were likely to be experienced by communities in Kenya and other similar settings:

In my opinion I think he [the requestor] should be given [data] because the benefits from research are for now and in the future, and so if his research will lead to … good things … then that will be a benefit to us as Kenyans. (R6FGD6, KCR)

In all groups, efficiency arguments for data sharing included reducing duplication and making better use of resources. These were seen by researchers as particularly important in sub Saharan Africa, given the relatively limited availability of research resources, such as manpower, skills, equipment and funding. More research-experienced participants also noted efficiencies in increased generalisability through comparisons of datasets, allowing wider skill sets to be drawn into the analysis of existing data and improving standardisation:

[Data sharing is] giving the proteomics world an opportunity to standardise things and in that way we’ll probably improve. (IDI02, researcher, Kenyan national)

Where data sharing limited unnecessary duplication of research, some researchers, health providers and other research staff anticipated reduced burdens of participation for those potentially involved in studies. Relatedly, a researcher felt there would be an expectation from participants that the ‘best use’ would be made of contributions to a study. Other community stakeholders *questioned a net benefit* of data sharing to study participants, since the benefits of participation would also be lost, recognising that research often supports availability of care where these occur together:

… if they [a putative primary research community] had participated themselves, wouldn’t they have benefited? … Somebody comes in, is ill, some research is done on them, but at the same time they get clinical care… are they going to lose out?’ (R1FGD2, field worker)

Several researchers noted data sharing as part of a societal trend towards increased open access to data, including for the Kenyan government [39] and in high income countries [40]. As a caution, a senior researcher emphasised the importance of evaluating data sharing overall and for specific datasets:

You may find out after doing this for five years, no one really cares! There’s some bloke in the north of Seattle who downloads every dataset, but apart from him…we are wasting our time trying to get the public interested in public health! (IDI06, researcher, UK national)

### Challenges and concerns for primary communities

Challenges and concerns about data sharing were consistently identified by stakeholders, however views about the nature and importance of the challenges varied within and across stakeholder groups. An overarching issue was the common perception that it would be difficult to generalise about the nature of potential risks and benefits since the type of data (noted particularly by researchers) and context (noted by all groups) would be important influences, underlining the importance of adequate governance.

### Risks of harm: Stigmatisation

The most fundamental issue, agreed by all stakeholders, was that data should not be shared in ways that might lead to harm for study participants or primary communities. Harms would be unacceptable in their own right and carry a risk of undermining researcher-community relationships in the long term, a feature of particular concern to researchers in Kilifi given their reliance on a long standing relationship with a given geographic community [41].

Across participants, the main potential harm seen for primary communities was stigmatisation where individuals or groups were identified and associated with potentially sensitive data. These concerns were largely addressed where stakeholders became aware of routine processes of de-identification (removal of all personal identifiers and replacement with codes). A specific challenge was the use of individual or group geo-positioning data, including village names:

The information…sometimes stigmatises some communities. Like there was once a cholera outbreak in a certain place and I heard some public health technicians saying people from that place are not clean. So… if you’re coming from that area, you feel bad! (R3FGD1, field worker)

While this data is important in some types of research, and identifying groups with a high prevalence of potentially stigmatising conditions could be a first step to addressing the underlying health condition, these concerns underpinned agreement that sharing data on unique characteristics or geographic locations of individuals or groups needed particular protection.

Identifying which data carried particularly sensitivities was difficult as this was commonly seen as related to its use, and likely to vary between individuals. The strongest but not universally agreed candidate was HIV status:

I think they are not that shocking if you like… I’m sure I would have answered this question differently if I was asked five or six years back… there’s been a lot of development in terms of HIV. (IDI10, researcher, Kenyan national)

In general, clinical information was seen as having potential sensitivities, including information about a person’s illness, diagnosis and management. Researchers and health providers saw data on genetics/genomics, sickle cell disease status, gender violence, and sexual exploitation and orientation as sensitive. Additional sensitive areas identified by community stakeholders (staff and non- staff) included information on sexual orientation and pregnancy status (the latter routinely collected in the Kilifi HDSS) and on socio-economic indicators (such as sanitation, education and literacy).

In addition to seeing associated risks of stigmatisation, all researchers and health providers perceived confidentiality as an important condition in its own right.

### Fairness to the primary community

As already noted, a key reason for sharing research data was the potential to generate short and long-term translational health benefits, that is, improvements in health policy and practice. While many community stakeholders initially sought local translational benefits, more research-experienced stakeholders often identified a wider public as the important target. A ‘public’ nature of benefit was always important, sometimes linked to the publically funded nature of much health research [42]:

So I guess my hard line would be, if… at the outset it’s not clear that [sharing] this data is going to benefit the wider public, then… personally I have a problem there. (IDI03, researcher, Kenyan national)

Amongst all groups, arguments generally developed to become less prescriptive about which community might benefit, for example, through recognising that common patterns of health problems and key scientific questions would change over time. Humanitarian arguments often supported this wider view:

For example a vehicle might be involved in an accident and people might be injured…At the hospital, stored blood is used to help those in need. So [research data] should be shared as those assisted are human beings too… (R6FGD4, assistant chief)

A potential for data sharing to generate public benefit was sometimes seen as satisfied by the involvement of international institutions representing public interests, such as the World Health Organization. In addition, an underlying assumption for many of the more research-experienced stakeholders was that the primary research had been designed in such a way that individual participants and their communities had already benefitted sufficiently:

So for me I wouldn’t really say that it must [benefit the local community] because I think the level where it should have benefitted them … was the reason why the data was collected in the first place. (R5FGD3, community facilitator)

The focus on primary communities’ interests supported suggestions that requests with greater local relevance should be prioritised; and that close collaborations should be built with local health policy makers to maximise opportunities for local or national short-term translational benefits. Collaborations could be through proactive data sharing, building skills for data use and developing research agendas in partnership. Notably, this was seen as a way of strengthening the ‘benefits side’ of a potential benefit/risk equation in data sharing:

If you’re a bit short on benefits then you tend to focus on risks…if we took more proactive steps to ensure benefits… I think we would have an easier time weighing [these]. (IDI07, researcher, UK national)

In addition, many researchers felt that primary communities should be recognised in future publications based on shared data, and that long-term benefits to primary communities could come from efforts to build capacity of national researchers.

### Challenges and harms for originating researchers

Amongst researchers and health providers, there was strong concern that originating researchers’ career development could be undermined by requirements for early data sharing, and support in principle for the use of embargo periods during which researchers need not share data [1]:

The data becomes more precious…when you feel that giving up that data might dampen your publicity, or slow your career development. (IDI02, researcher, Kenyan national)

She knew we were collecting data over here and she gave the impression that she could just get all the data and publish it and then I told her, “Well I have been the one collecting it with the intention of publishing”… She looked too keen to analyse the data without having been that involved in collection. (IDI09, researcher, Kenyan national)

Primary researchers often articulated their concerns through reference to ownership (‘my data’), linked to their investment of resources in generating databases. Claims of ownership also functioned to control data use in future, including strengthening scientific validity and preventing types of ‘misuse’. However embargo periods were not seen as straightforward to implement, given risks of researchers being unreasonably protective and over-estimating their capacity to utilise data fully.

The interests of originating researchers became more clearly fixed and agreed for those working in less well-resourced settings. The focus of concern was on the relative abilities of researchers in different parts of the world to harness the technical expertise needed for data analysis. This challenge was not therefore limited to low-to-middle income countries, but to research institutions with limited access to resources:

If it’s open access, someone else who has the skills that you may not necessarily have at the moment could very quickly do an analysis that you’d want to do… someone in Harvard could walk very quickly to a next door neighbour and get that analysis done overnight and published… (IDI02, researcher, Kenyan national)

Many community stakeholders similarly felt that originating researchers had a reasonable vested interest in the data they collect, expressing concern that local researchers’ careers should not be ‘overtaken’ by others who had made less investment.

Originating researchers could also be undermined when new analyses appeared to contradict original work. This issue—the flipside to a benefit of ensuring reproducibility—was seen as challenging to a collegiate view of science and likely to generate reluctance to share data, particularly amongst researchers with least access to resources:

Sharing of data should just clearly be seen as a productive way of getting new knowledge, but not hammering, or not saying that your methods are not working, or maybe not degrading. It should be … emphasized that it is a healthy activity. (IDI10, researcher, Kenyan national)

Many researchers with experience of sharing data as originators or members of research governance groupings in the programme described well-recognised challenges in mobilising resources to support data sharing, including for cleaning and storing data in ways that support sharing, and providing governance of future data access requests [23]. Particular resources described were data management and governance skills and funding support.

### Misuse of data

In all groups there were very general concerns about a potential for data sharing to lead to intentional or unintentional ‘misuse’ of data. Unintentional misuse included requestors using data without sufficient understanding of the data or its context, with important risks for science. This risk emphasised a need to provide sufficient metadata to describe data; to seek greater standardisation; and to encourage communication between requestors and originating researchers to limit misinterpretation. Intentional misuse was spoken about in all groups, very generally and as a potential risk rather than a problem experienced. The term fundamentally implied use in ways not initially agreed or understood, and reflected recognition of high levels of uncertainty about how data might be used once it had been shared, including misrepresentation, use for other purposes and passing data on to third parties.

For community stakeholders, misuse was mainly described as forms of exploitation of the primary community. For example, a KCR made links to rumours of researchers ‘stealing’ knowledge about traditional medicine to use for commercial purposes. A community facilitator described loss of trust when a private consultant ‘stole’ ideas from a community development group to develop a proposal of his own. The main feature of these concerns was a lack of control of how data could be used after sharing, and the importance of trust, including institutional forms, in these situations:

When KEMRI started and collaborated with Kilifi hospital…that’s when we knew there is KEMRI. But for [data requestor]…we have to know where he is from, what is his relationship with the government, is he in collaboration with the hospital too? We haven’t known anything, how are we going to work with him? (R3FGD5, KCR)

Many community stakeholders gained confidence in proposals for data sharing as concepts (such as anonymisation) and purposes were clarified, and misunderstandings and concerns discussed. But issues related to misuse—given its characteristic of uncertainty—were not straightforward to address.

Researchers and health providers similarly identified a problem of intentional misuse linked to uncertainties in data sharing that routine governance approaches would be unlikely to mitigate. Ultimately, primary researchers and communities must take on trust that a requestor given information would not exceed the terms of any data sharing agreement. Anonymising data was seen to decrease but not exclude risks of misuse. A common response to these concerns was an expressed preference for sharing data with other known researchers, including within collaborations.

At the same time, a significant number of researchers—particularly those with strongly positive attitudes towards data sharing—felt that risks of harms or misuse were routinely over-estimated. Relatedly, it was felt that non-specific concerns about misuse might be linked to an underlying discomfort about the balance between benefits and risks, as described earlier.

### Does it matter who’s asking?

For many community stakeholders, the importance of a requestor’s identity mapped on to questions of local translational benefits; the further away from Kilifi a requestor was, the less likely that local benefits would follow. In addition, increasing distance implied less connectivity and therefore trust in how a researcher might use data, particularly for requestors outside Kenya. While views on the importance of short-term translational benefits for primary communities became less prescriptive over time, issues of trust and concerns about potential for misuse by non-national requestors remained important for community stakeholders. For these reasons, most emphasised a need for national-level government representation in decisions about sharing data outside Kenya:

So if we say that this organisation… is requesting data from KEMRI … still it should go to the government to explain its work… (R6FGD5, KCR)

Researchers and health providers emphasised the importance of sharing data with local and national Ministry of Health colleagues to promote national and local translational benefits; and described greater comfort with sharing data within established scientific collaborations. For the latter, communication would be easier, including clarifications about data; capacity building within the programme would be more likely; and risks of unintentional and intentional misuse less. Researchers and health providers were less concerned about sharing data internationally than community stakeholders given the international nature of existing scientific collaborations.

In this way, across all groups, the importance of taking account of the relationships involved in data sharing emerged as a key issue. Illustratively, a ‘convention of practice’, including forms of reciprocity, was seen as important:

I prefer that there be…a convention of practice about utilising data… some sort of co-operative forum in which people can come in and say, “I’d like to use this piece of data. I know you collected it…I’ve got this really good idea, could we do something together? (IDI04, researcher, UK national).

Reciprocity emerged across many researchers’ account of fair practice, for example, as reluctance to share data with those who choose to ‘ride on other’s work’ rather than collect data of their own; and as an increased willingness to share data when there is a concomitant need to access others’ data.

Separate to issues of trust, several researchers expressed an underlying preference for sharing data with researchers working within sub Saharan Africa, based on seeing long-term benefits in building local scientific capacity as well as increased public acceptability of data sharing:

I think the use by researchers within sub Saharan Africa [of data collected in this region] …makes using data more acceptable to participants… its part of the idea that you build capacity within the region to use its own data. So…to get top people in the US to analyse all the data is a very short-term strategy, whereas a long-term strategy is to develop capacity locally. (IDI07, researcher, UK national)

## Discussion

An important component of developing responsive policies for public health data sharing, institutionally, nationally and internationally, is taking account of the views and values of research stakeholders in a given context. Taken overall, these findings constitute a valuable contribution to understanding views in a particular context, and address a gap in the literature in bringing the voices of different types of research stakeholders to the existing debate, that is, public voices and those from LMIC settings. Many perceptions of benefits and challenges for primary communities and originating researchers arose in all stakeholder groups, often in a highly interrelated form, supporting arguments (made for genomic data) that ethical analyses of data sharing should consider the ‘complex interplay of stakeholders and their interests, rather than single-issue and single-stakeholder perspectives’(p633) [12]. In this discussion, we highlight findings reflecting existing or new issues in the literature, emphasising contextual influences in a LMIC setting, and explore the strongly emerging importance of trust in data sharing practice and policy. In a separate paper, we discuss these implications for institutional policy and practice [23].

Throughout this consultation, it has been important to take account of limitations that may be related to the methodology used. Deliberative forms of public consultation can generate particular challenges in influencing views expressed, particularly through ‘biases’ implicit in the attitudes of facilitators and within-group dynamics [43, 44]. At the same time, information sharing and the development of informed viewpoints over time, including through debate, have formed an essential basis for this consultation, and added to our understanding of risks related to partial forms of understanding. As described in the methods section of the paper, throughout the planning and conduct of the study, we maintained high awareness of and sought to limit these influences.

### Acceptability of data sharing and contextual influences

Nearly all more and less research-experienced stakeholders accepted or expressed positive attitudes to research data sharing, while at the same time describing a range of concerns and underlying prerequisite conditions. Oversight for policies and access decisions that take account of these conditions are therefore likely to be an important feature of ethical data sharing practices. If forms of governance can be developed with support from diverse stakeholders, data sharing is likely to be an acceptable or even valued component of research in this setting. Community engagement and informed consent will be key to a responsive process, as are discussed in more detail elsewhere [23].

In general, researchers, health providers and community facilitators were more strongly positive than other stakeholders. To some extent, caution amongst less research-experienced stakeholder groups reflected incomplete understanding of the nature of data and of protections that could be put in place, and often became less marked through the process of information sharing. At the same time, many stakeholders consistently expressed concerns about data sharing, strongly highlighted by the nature of prerequisite conditions described. For researchers and health providers, the benefits of data sharing reflected those in the literature, including strengthening reproducibility and efficiency, and reducing burdens to participants in research [15, 42]. In this consultation, while similar benefits were seen by community stakeholders, this group also noted that reducing ‘burdens’ of participation by cutting back on duplication of studies may limit ‘benefits’ for potential participants. The forms of study benefit ‘lost’ may be particularly important in LMICs, notably where clinical research contributes to strengthening health services [41].

Global inequities in access to scientific resources acted as a prompt to concerns about the *interests of originating researchers* in international data sharing [1, 11, 13, 18] in addition to more general risks [11, 14, 16]. Allowing better resourced scientists to gain relative advantages over those working in more constrained circumstances was seen as having potential short-term gains for science, but undermining long-term progress towards closing gaps and building scientific capacity internationally [13, 20]. Our findings suggest additional risks of losing public trust in less well-resourced settings. These findings support arguments that, since scientific capacity in LMICs is likely to be fundamental to long term improvements in health, undermining progress in science may also impact health service delivery and ultimately public health in these settings [19, 20].

In relation to primary community interests, a fundamental condition for data sharing across all groups was that primary communities should not been harmed. This was seen as both intrinsically wrong and instrumentally problematic for trust in researcher-community relations. Loss of privacy was similarly seen as intrinsically wrong and as generating risks of stigmatisation when associated with sensitive data. In general, the *use* of data rather than the nature of data was linked to potential sensitivities, but many community stakeholders perceived risks that socio-economic data could be used in disrespectful or stigmatising ways. The sensitivities identified by researchers mirrored those in the literature, including for clinical data, particularly HIV status, genomic data and data on sexual behaviour; although it was recognised that sensitivities often change over time. These findings underline the importance of oversight for access to data, given challenges in identifying (and applying special protections to) ‘sensitive’ data *per se* and the importance of monitoring processes over time.

Less well described in the literature, there was widespread concern about fairness towards primary communities. Fairness was mainly expressed as ensuring sufficient translational benefits for health from data sharing, either locally (by community stakeholders) or for wider publics (by all). In particular, increased near-term translational benefits from data sharing would strengthen the overall risk-benefit balance for participants. For researchers, recognition of underlying global health inequities in health and access to health resources often underpinned these concerns. While local translational benefits of data sharing could be trumped by wider interests of science, many researchers continued to feel a responsibility to work in ways that diminished, or at least did not increase, underlying inequities. This view seems to widen the scope of issues and stakeholders relevant to the ethics of data sharing in several ways [12]. Firstly, it implies that fairness in benefit sharing arrangements in primary research [35] are put into particular focus when data will be shared. Secondly, it highlights the importance of researchers supporting near-term local translational benefits through strong partnerships with government health policy makers, including through developing joint agendas for research. Global forms of inequity are well recognised in the literature as an important influence on ethical research policy [45, 46]. Based on our findings, data sharing policies may be influenced in the same way, in relation to the interests of both originating researchers and primary communities.

### Trust and social relations in data sharing

Across all stakeholder groups, many concerns centred on inherently unknowable variables in data sharing, including unknown requestors and purposes and timing of requests, generating a central importance of trust in stakeholder relations [47]. As before, in this LMIC setting, trust was fundamentally linked to confidence that originating researchers’ and primary communities’ interests *would be not only protected but as far as possible promoted during processes of data sharing*.

In this consultation, where researchers were in the most part more comfortable sharing data with known requestors or networks, scientific collaborations emerged as important trust-building mechanisms. Collaborations were seen to provide originating researchers more control over how data were used and in leveraging reasonable levels of reciprocity [1, 9, 16].

For many community stakeholders in this consultation, sharing research data with requestors outside national boundaries evoked similar questions of trust, based on a relationship between unknowns and degrees of geographic connectivity. These views mirror findings on community stakeholders’ perspectives on sharing biological samples in Kenya and Ghana [48]; community stakeholders’ views on data sharing in Canada [21] and a commentary based on experience of sharing data from indigenous communities in New Zealand [9]. For many community stakeholders in this consultation, the concept of data itself was difficult to grasp, heightening uncertainties about what risks data sharing might incur. Similar issues were described for community stakeholders in India, as part of the PHRDS multi-country project [24]. Given these concerns, the central recommendation for practice in Kenya was that governance of international data sharing should take place within a clear national framework [20].

Public attitudes to the KWTP, and the existing relationship between local residents and research staff, are likely to colour views about research policy and practice, such as proposals for data sharing. As described earlier, KWTP is a relatively long standing institution with an active community engagement programme. Within sections of the community, we are aware of appreciation of the research programmes’ role in providing local employment, supporting medical services through partnership with county health managers, providing locally responsive study benefits to participants and conducting research seen as relevant [35, 41]. The context for this study is therefore likely to have an important influence on the findings and our conclusions. In our setting, trust can be seen as important across wider aspects of the researcher-study participant/community relationship than that related to data sharing policy only. The need for institutional-wide policies that are trust-building is examined in more detail in a separate publication, focusing on areas important for data sharing [23]. In outline, areas of importance include building community-wide awareness and understanding of data sharing purposes and means; developing appropriate informed consent processes; involving community members in developing data sharing policy; providing community accountability for access decisions taken; and promoting data sharing through scientific collaborations and within a national framework.

## Conclusions

For more and less research-experienced stakeholders, ethical research data sharing is likely to rest on the development and implementation of appropriate trust-building mechanisms, linked to local perceptions of benefits and challenges. Key benefits in this consultation concerned the promotion of public health through science, with legitimate beneficiaries defined differently by different groups. Important challenges were risks to the interests of study participants, communities and originating researchers through stigmatisation, loss of privacy, impacting autonomy and unfair competition, including through forms of intentional and unintentional ‘misuse’ of data. Risks were also seen for science. Inherent uncertainties in data sharing (about what data will be shared with whom, for what purpose and when) meant that building trust in relationships within and between stakeholder groups was a key component of ethical practice. Given background structural inequities in much international research, building trust in this low-to-middle income country setting includes ensuring that the interests of study participants, primary communities and originating researchers will as far as possible be promoted as well as protected. Mechanisms to support trust building include public involvement in policy development and implementation, promoting scientific collaborations around data sharing, and building close partnerships between researchers and government health authorities to provide checks and balances on data sharing, and promote near and long-term translational benefits.

Information sharing will be needed to support community awareness and individual informed consent processes for data sharing. These are not trivial requirements. Our findings underline both the possibilities and challenges of building understanding of many technical aspects of data sharing, including the nature of research, the nature of data and how its secondary use might be of value. Significant resources, including communication skills and supportive community-researcher relationships, will be needed for these forms of engagement.

In reaching these conclusions, we recognise that the context for this consultation, and for research in KWTP, are likely influences on our findings. It is important to assess perspectives on benefits and challenges of data sharing in different settings to understand the transferability of these findings, a process begun through the PHRDS multi-country project that this study emerges from [49–53]. It will be important to assess these over time given the relative novelty of data sharing for many research stakeholders. At the same time, drawing on the wider literature and our findings, it is highly likely that trust and the nature of relationships between researcher or research institutions and primary communities of different types will remain a constant influence on attitudes to research data sharing, and an important determinant of ethical policy [24, 25]. As Foster and Sharp (2007) point out, data sharing is ‘as much a social as a scientific question’ (p638) [12].

## Supporting Information

S1 Document Information used in vignette and question guide for community stakeholders’ discussions. (DOCX)

S2 Document Focus Group Discussion topic guide. (DOCX)

S3 Document In-depth Interview topic guide. (DOCX)
