# Supplementary material for: Research Stakeholders’ Views on Benefits and Challenges for Public Health Research Data Sharing in Kenya: The Importance of Trust and Social Relations
Source: PLoS One. 2015 Sep 2;10(9):e0135545. doi: 10.1371/journal.pone.0135545 (PMC4557837; doi:10.1371/journal.pone.0135545)
Supplement: S1 Document — (DOCX) [file pone.0135545.s001.docx]

**S1 Doc. Information used in vignette and question guide for community stakeholders’ discussions**

A researcher working with another government research institution in Kenya needs data to try to find out if there is any association between having good access to water supplies and chances of being admitted to hospital for diarrhoea. Results from this work will help to come up with better ways of preventing diarrhoeal diseases in the community the researcher is working with. The researcher is working in an area which is very like Kilifi but do not have a census or hospital surveillance system. The information s/he is requesting is: i) all clinical information collected about individuals admitted to hospital in the last year (explain what this is); ii) what kind of water supply they have at home (from census).

Should this information be shared? Why/why not? What, if anything, could be done to make data sharing more acceptable?

What if the researcher is working at another institution/non-governmental organization?

What if they come from outside Kenya/outside Africa?

What if they want the information for research on health conditions not common in Kilifi/Kenya?
